# Supplementary figures and images for: Recovering or Persisting: The Immunopathological Features of SARS-CoV-2 Infection in Children
Source: J Clin Med. 2022 Jul 27;11(15):4363. doi: 10.3390/jcm11154363 (PMC9369242; doi:10.3390/jcm11154363)

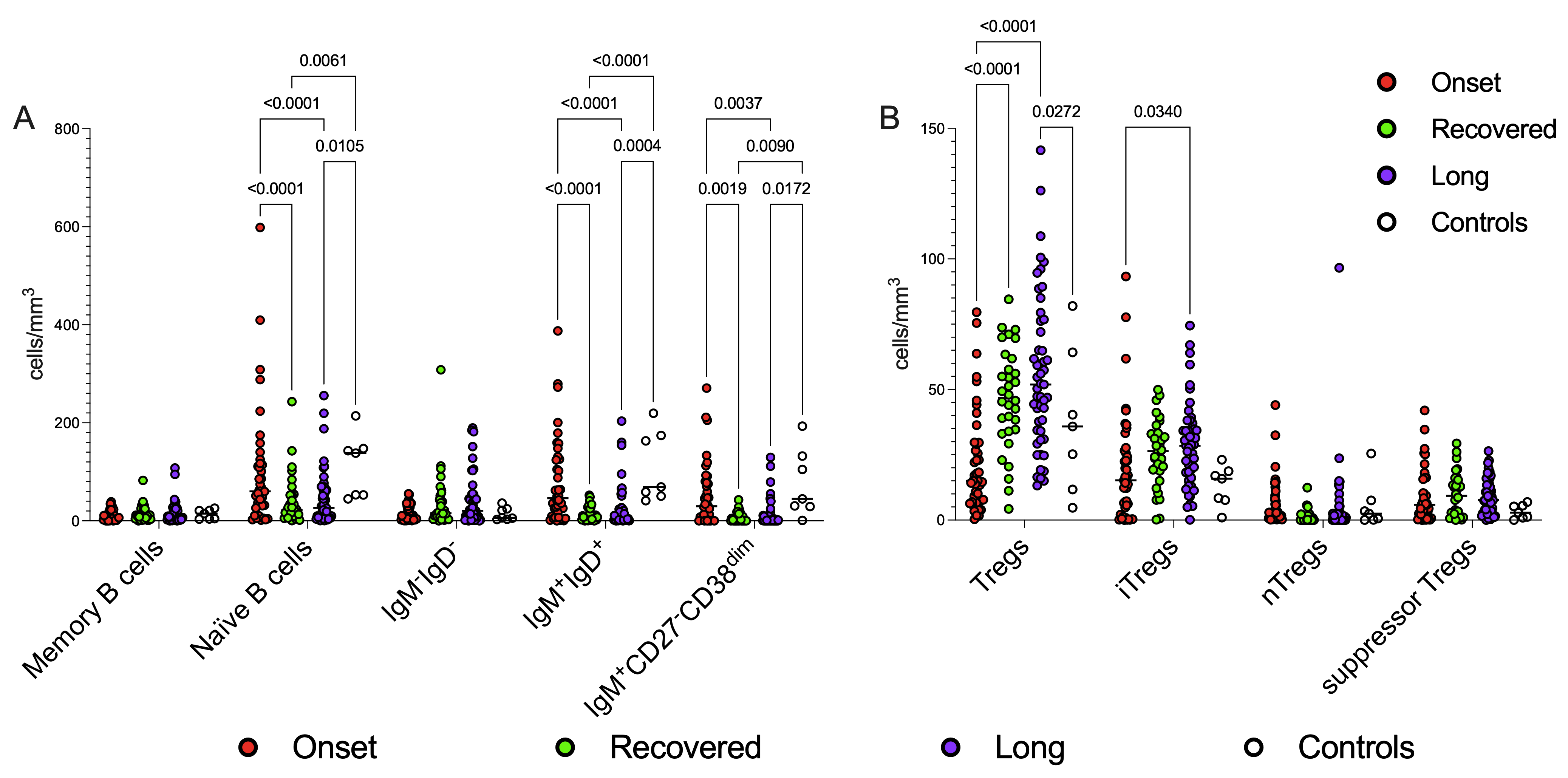

Supplement: Supplementary file 1 [file jcm-11-04363-s001.zip › Figure S1.tiff]

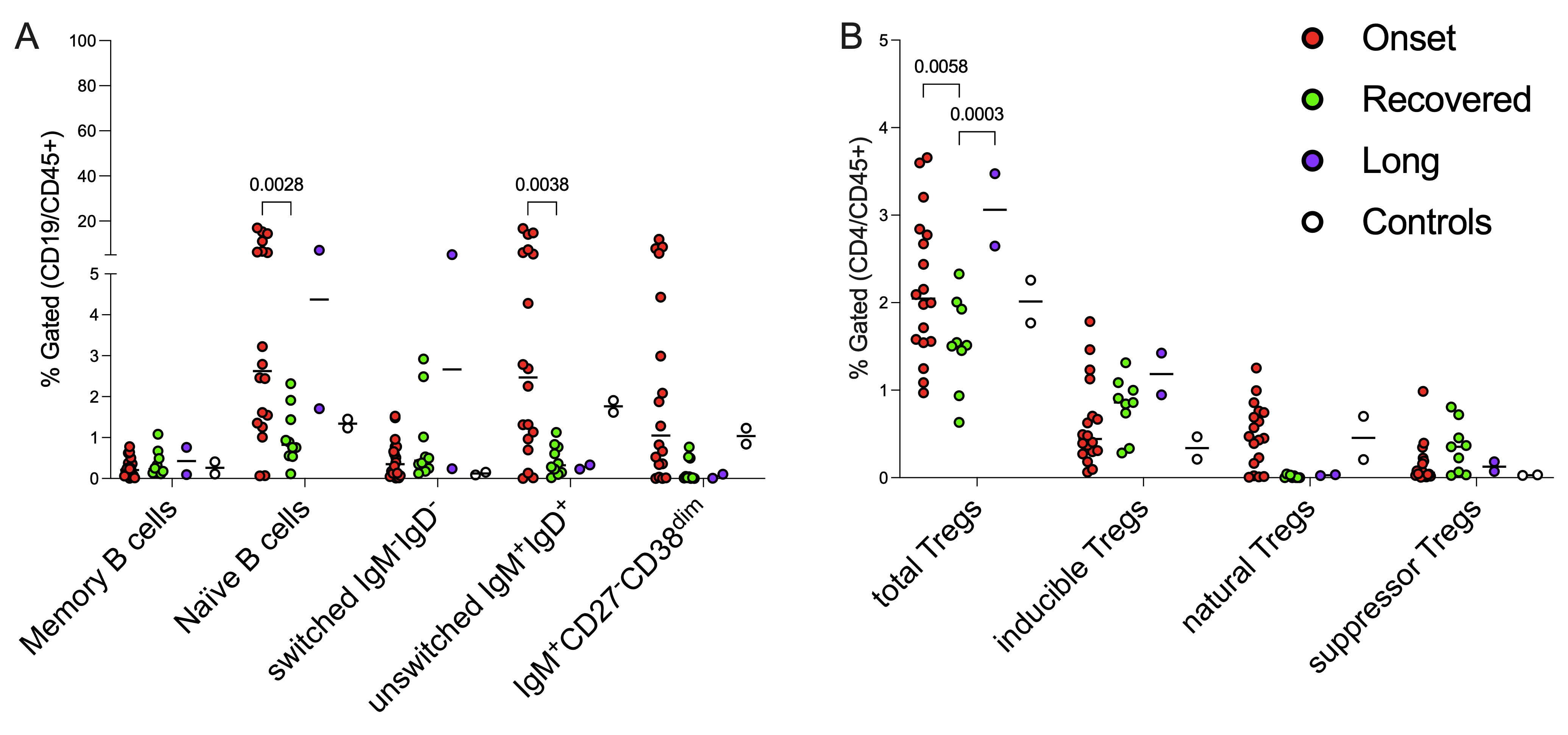

Supplement: Supplementary file 1 [file jcm-11-04363-s001.zip › Figure S2.tiff]
